# Crystal structure of 4-[(5-methyl­isoxazol-3-yl)amino­sulfon­yl]anilinium 3,5-di­nitro­salicylate

**DOI:** 10.1107/S2056989015008701

**Published:** 2015-05-13

**Authors:** Sevaiyan Malathy, Jeyaraman Selvaraj Nirmalram, Packianathan Thomas Muthiah

**Affiliations:** a School of Chemistry, Bharathidasan University, Tiruchirappalli 620 024, Tamil Nadu, India

**Keywords:** crystal structure, sulfamethoxazolium, 3,5-di­nitro­salicylate, mol­ecular salt, hydrogen bonding.

## Abstract

The title mol­ecular salt, C_10_H_12_N_3_O_3_S^+^·C_7_H_3_N_2_O_7_^−^, protonation occurs at the amino N atom attached to the benzene ring of sulfamethoxazole. In the anion, there is an intra­molecular O—H⋯O hydrogen bond and the cation is linked to the anion by an N—H⋯O hydrogen bond. In the extended structure, the cations and anions are linked *via* N—H⋯O, N—H⋯N and C—H⋯O hydrogen bonds, forming a three-dimensional framework.

## Abstract 2

The title mol­ecular salt, consists of a sulfamethoxazolium (SMZ) cation and a 3,5-di­nitro­salicylate (DNS) anion, which are linked by an N—H⋯O hydrogen bond. In the crystal, the cations and anions are linked *via* N—H⋯O, N—H⋯N and C—H⋯O hydrogen bonds, forming a three-dimensional framework.

## Chemical context   

Sulfamethoxazole, {4-[(5-methyl­isoxazol-3-yl)amino­sulfon­yl]aniline} (SMZ) is a well-known anti­bacterial and anti­fungal sulfa drug (Ma *et al.*, 2007 ▸; Hida *et al.*, 2005 ▸). This drug prevents the formation of di­hydro­folic acid, a compound that bacteria must be able to make in order to endure. The structural resemblance of *p*-amino benzoic acid to the sulfanilamide group enables sulfanilamide block folic acid synthesis in bacteria (Bock *et al.*,1974 ▸). SMZ is also known to be effective against gram positive and gram negative bacteria and some protozoans. In clinical practice, SMZ is used as a combinatorial drug along with Trimethoprim (TMP) to treat a variety of bacterial infections. In the last three and half decades, multiple crystalline forms of SMZ (Bettinetti *et al.*, 1982 ▸; Maury *et al.*, 1985 ▸; Price *et al.*, 2005 ▸), metal complexes (Marques *et al.*, 2006 ▸; Nakai *et al.*, 1984 ▸) and salt forms (Nakai *et al.*, 1984 ▸; Subashini *et al.*, 2007 ▸) have been reported. We report herein on the crystal structure and supra­molecular packing pattern of the title salt.
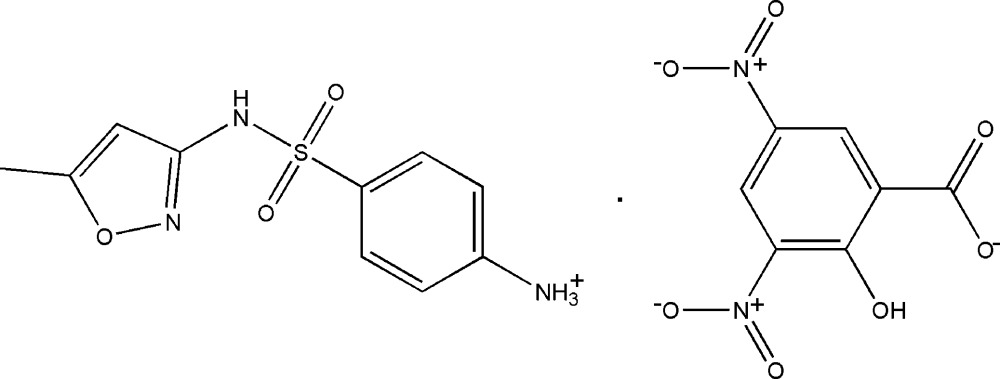


## Structural commentary   

**Figure 1:**
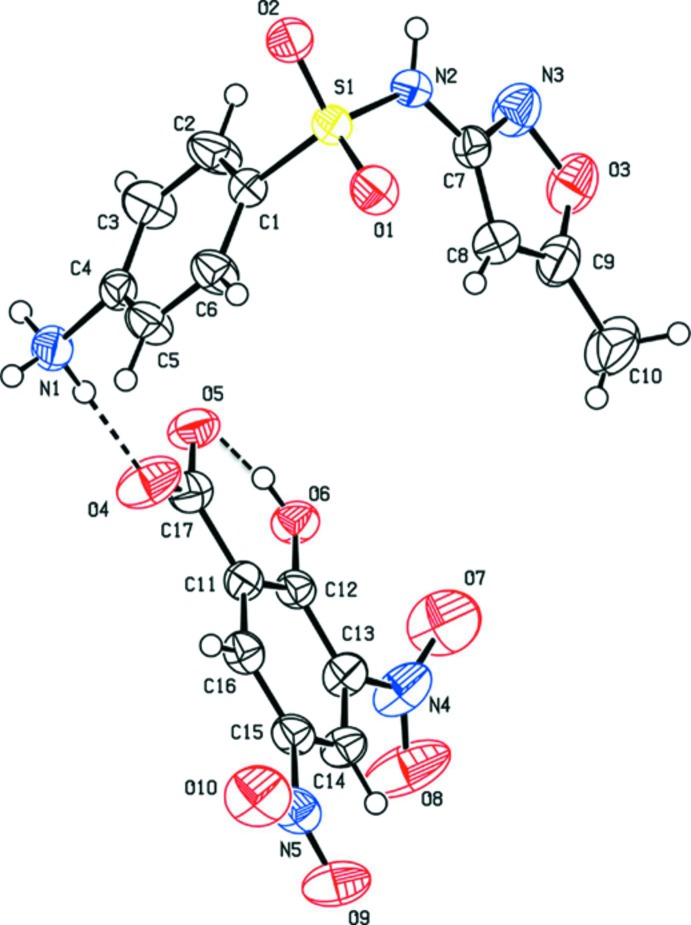
A view of the mol­ecular structure of the title mol­ecular salt, showing the atom labelling. The displacement ellipsoids are drawn at the 50% probability level. The hydrogen bonds are shown as dashed lines (see Table 1 ▸ for details).

The asymmetric unit of the title salt (SMZDNS), consists of a sulfamethoxazolium cation and a 3,5-di­nitro­salicylate anion (Fig. 1 ▸). The SMZ cation is L-shaped with the dihedral angle between the oxazole and anilinium rings being 81.86 (10)°. The geometry around the sulfur atom is slightly distorted tetra­hedral, which is evident from the O1—S1—O2 angle of 120.44 (8)°. Protonation occurs at the amino atom N1 of the benzene moiety of SMZ. In the cation there is an intra­molecular O—H⋯O hydrogen bond with an *S*(6) ring motif (Fig. 1 ▸ and Table 1 ▸). The cation is linked to the anion by an N—H⋯O hydrogen bond (Fig. 1 ▸ and Table 1 ▸), and the dihedral angle between the benzene rings of the cation and anion is 78.51 (8)°.

## Supra­molecular features   

**Figure 2:**
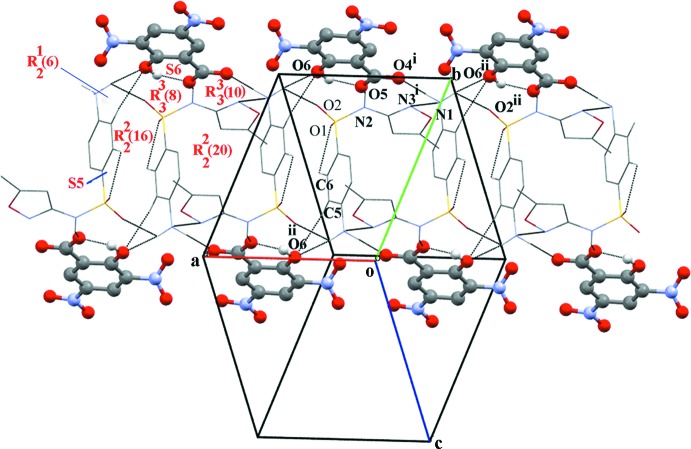
A view of the graph set motifs formed in the crystal of the title salt, *via* N—H⋯O, N—H⋯N and C—H⋯O hydrogen bonds (dashed lines; see Table 1 ▸ for details). The cations are drawn in wire mode and the anions in ball-and-stick mode.

In the crystal of the title salt, there are various hydrogen bonds present linking the anions and cations and forming a three-dimensional network (Figs. 2 ▸ and 3 ▸, and Table 1 ▸). The ammonium ion of the cation generates a *C*(3) chain and two 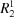
(6) and 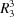
(10) ring motifs (Bernstein *et al.*, 1995 ▸). The primary inter­action between the cation and anion happens through an N—H⋯O hydrogen bond and it forms a chain of *C*(3) graph set. The 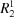
(6) motif is formed *via* N—H⋯O and C—H⋯O hydrogen bonds that link the ammonium N1 phenyl C5 group of SMZ and the hy­droxy O6 group of the anion. The 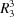
(10) ring motif is a result of the linking of two symmetry-related cations and one anion *via* a pair of N—H⋯O and N—H⋯N hydrogen bonds. This motif is formed by the inter­action of symmetry-related imino N2, oxazole N3, ammonium N1 atoms of the cation and the carboxyl­ate (O4 and O5) group of the anion. The 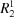
(6) and 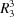
(10) motifs are linked by another ring motif with an 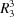
(8) graph set. This motif is formed by linking two symmetry-related cations with an anion *via* a pair of bifurcated N—H⋯O hydrogen bonds. The amalgamation of the above ring motifs leads to the formation of supra­molecular sheets along the *a* axis (Fig. 2 ▸). The sheets thus formed are linked to adjacent ones through 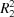
(16) and 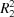
(20) motifs. The 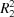
(16) motif is formed by inter­action of ammonium atom N1 and atom O2 of the sulfate group of an inversion-related SMZ ion in an adjacent sheet *via* a pair of N—H⋯O hydrogen bonds. The other motif, an 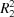
(20) ring, is formed by the linkage of two inversion-related cations along the *b* axis. Finally, through these arrangements a three-dimensional hydrogen-bonded architecture is formed.

## Database survey   

A search of the Cambridge Structural Database (Version 5.36; Groom & Allen, 2014 ▸) for 4-[(5-methyl­isoxazol-3-yl)amino­sulfon­yl]aniline revealed the presence of only two structures of the protonated form. These include, *catena*-[bis­(sulfa­methoxazolium)(μ2-chlorido)­tri­chlorido­cadmium(II) monohydrate] [RISZAV; Subashini *et al.*, 2008 ▸] and 4-[(5-methyl­isoxazol-3-yl)amino­sulfon­yl]anilinium chloride (also known as sulfamethoxazole chloride; SIMJEE; Subashini *et al.*, 2007 ▸). The dihedral angles between the oxazole ring and anilinium ring is found to be *ca* 88° in RISZAV, similar to the value of 81.86 (10)° in the title salt, and *ca* 58° in SIMJEE.

## Synthesis and crystallization   

20 ml of a hot ethano­lic solution of sulfamethoxazole (63 mg) and 3.5 di­nitro­salicylic acid (57 mg) were mixed and warmed at 323 K for 30 min over a water bath. The mixture was then allowed to cool slowly at room temperature. Three weeks later, light-yellow prismatic crystals were obtained.

## Refinement   

Crystal data, data collection and structure refinement details are summarized in Table 2 ▸. All H atoms were positioned geometrically and refined using a riding model: O—H = 0.82 Å, N—H = 0.86–0.89 Å, and C—H = 0.93–0.96 Å with *U*_iso_(H) = 1.5*U*_eq_(C,O,N) for methyl, hy­droxy and ammonium H atoms and 1.2*U*_eq_(C,N) for aromatic and other H atoms.

## Supplementary Material

Crystal structure: contains datablock(s) global, I. DOI: 10.1107/S2056989015008701/su5130sup1.cif

Structure factors: contains datablock(s) I. DOI: 10.1107/S2056989015008701/su5130Isup2.hkl

Supporting information file. DOI: 10.1107/S2056989015008701/su5130Isup3.cml

CCDC reference: 1063245

Additional supporting information:  crystallographic information; 3D view; checkCIF report

## Figures and Tables

**Figure 3:**
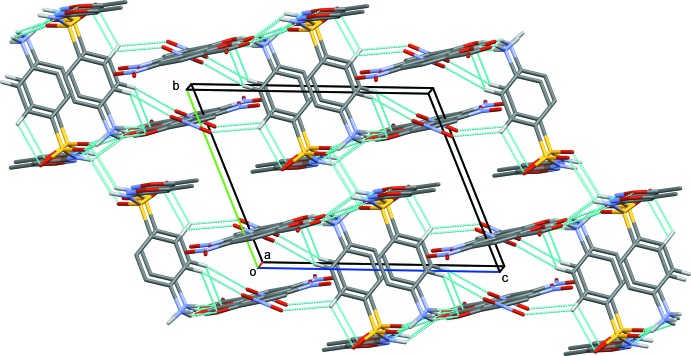
A view along the *a* axis of the crystal packing of the title salt. The hydrogen bonds are drawn as dashed lines (see Table 1 ▸ for details). H atoms not involved in hydrogen bonding have been omitted for clarity.

**Table 1:** Hydrogen-bond geometry (, )

*D*H*A*	*D*H	H*A*	*D* *A*	*D*H*A*
O6H6*A*O5	0.82	1.68	2.4296(19)	151
N2H2*A*O5	0.86	2.12	2.7852(18)	134
N1H1*A*O4^i^	0.89	1.77	2.661(2)	177
N1H1*B*N3^i^	0.89	2.24	3.041(2)	150
N1H1*C*O6^ii^	0.89	2.21	3.064(2)	160
C5H5O6^ii^	0.93	2.60	3.293(2)	132
C6H6O8^iii^	0.93	2.60	3.176(2)	121

Symmetry codes: (i) 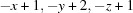; (ii) 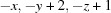; (iii) 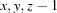.

**Table 2:** Experimental details

Crystal data	
Chemical formula	C_10_H_12_N_3_O_3_S^+^C_7_H_3_N_2_O_7_
*M*_r_	481.41
Crystal system, space group	Triclinic, *P* 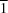
Temperature (K)	296
*a*, *b*, *c* ()	8.5551(1), 10.5000(2), 12.7576(3)
, , ()	106.463(1), 100.913(1), 108.272(1)
*V* (^3^)	993.72(3)
*Z*	2
Radiation type	Mo *K*
(mm^1^)	0.23
Crystal size (mm)	0.20 0.20 0.16

Data collection	
Diffractometer	Bruker Kappa APEXII CCD
Absorption correction	Multi-scan (*SADABS*; Bruker, 2004 ▸)
*T*_min_, *T*_max_	0.955, 0.964
No. of measured, independent and observed [*I* > 2(*I*)] reflections	24261, 6718, 4911
*R*_int_	0.030
(sin /)_max_ (^1^)	0.758

Refinement	
*R*[*F* ^2^ > 2(*F* ^2^)], *wR*(*F* ^2^), *S*	0.048, 0.139, 1.05
No. of reflections	6718
No. of parameters	301
H-atom treatment	H-atom parameters constrained
_max_, _min_ (e ^3^)	0.40, 0.40

Computer programs: *APEX2* and *SAINT* (Bruker, 2004 ▸), *SHELXS97* and *SHELXL97* (Sheldrick, 2008 ▸), *PLATON* (Spek, 2009 ▸), *Mercury* (Macrae *et al.*, 2008 ▸), *POVRay* (Cason, 2004 ▸) and *publCIF* (Westrip, 2010 ▸).

## supplementary crystallographic information

### Crystal data

**Table:**

C_10_H_12_N_3_O_3_S^+^·C_7_H_3_N_2_O_7_^−^	*Z* = 2
*M**_r_* = 481.41	*F*(000) = 496
Triclinic, *P*1	*D*_x_ = 1.609 Mg m^−^^3^
Hall symbol: -P 1	Mo *K*α radiation, λ = 0.71073 Å
*a* = 8.5551 (1) Å	Cell parameters from 6718 reflections
*b* = 10.5000 (2) Å	θ = 1.8–32.6°
*c* = 12.7576 (3) Å	µ = 0.23 mm^−^^1^
α = 106.463 (1)°	*T* = 296 K
β = 100.913 (1)°	Prism, yellow
γ = 108.272 (1)°	0.20 × 0.20 × 0.16 mm
*V* = 993.72 (3) Å^3^	

### Data collection

**Table:**

Bruker Kappa APEXII CCD diffractometer	6718 independent reflections
Radiation source: fine-focus sealed tube	4911 reflections with *I* > 2σ(*I*)
Graphite monochromator	*R*_int_ = 0.030
ω and φ scan	θ_max_ = 32.6°, θ_min_ = 1.8°
Absorption correction: multi-scan (*SADABS*; Bruker, 2004)	*h* = −12→12
*T*_min_ = 0.955, *T*_max_ = 0.964	*k* = −15→15
24261 measured reflections	*l* = −19→16

### Refinement

**Table:**

Refinement on *F*^2^	Primary atom site location: structure-invariant direct methods
Least-squares matrix: full	Secondary atom site location: difference Fourier map
*R*[*F*^2^ > 2σ(*F*^2^)] = 0.048	Hydrogen site location: inferred from neighbouring sites
*wR*(*F*^2^) = 0.139	H-atom parameters constrained
*S* = 1.05	*w* = 1/[σ^2^(*F*_o_^2^) + (0.0667*P*)^2^ + 0.2293*P*] where *P* = (*F*_o_^2^ + 2*F*_c_^2^)/3
6718 reflections	(Δ/σ)_max_ < 0.001
301 parameters	Δρ_max_ = 0.40 e Å^−^^3^
0 restraints	Δρ_min_ = −0.40 e Å^−^^3^

### Special details

**Table:**

Geometry. Bond distances, angles *etc*. have been calculated using the rounded fractional coordinates. All su's are estimated from the variances of the (full) variance-covariance matrix. The cell e.s.d.'s are taken into account in the estimation of distances, angles and torsion angles
Refinement. Refinement on *F*^2^ for ALL reflections except those flagged by the user for potential systematic errors. Weighted *R*-factors *wR* and all goodnesses of fit *S* are based on *F*^2^, conventional *R*-factors *R* are based on *F*, with *F* set to zero for negative *F*^2^. The observed criterion of *F*^2^ > σ(*F*^2^) is used only for calculating -*R*-factor-obs *etc*. and is not relevant to the choice of reflections for refinement. *R*-factors based on *F*^2^ are statistically about twice as large as those based on *F*, and *R*-factors based on ALL data will be even larger.

### Fractional atomic coordinates and isotropic or equivalent isotropic displacement parameters (Å^2^)

**Table:**

	*x*	*y*	*z*	*U*_iso_*/*U*_eq_	
S1	−0.02011 (5)	0.65153 (4)	0.38510 (3)	0.0339 (1)	
O1	−0.12139 (15)	0.56291 (12)	0.26954 (11)	0.0467 (4)	
O2	−0.09563 (16)	0.65425 (13)	0.47595 (11)	0.0464 (4)	
O3	0.47464 (18)	0.55503 (17)	0.32047 (14)	0.0620 (5)	
N1	0.29162 (18)	1.24424 (14)	0.38400 (13)	0.0425 (4)	
N2	0.13876 (17)	0.60279 (14)	0.42019 (12)	0.0379 (4)	
N3	0.3923 (2)	0.57678 (19)	0.40572 (15)	0.0531 (5)	
C1	0.07470 (18)	0.82911 (15)	0.38933 (13)	0.0317 (4)	
C2	0.2170 (2)	0.93167 (19)	0.47915 (16)	0.0503 (5)	
C3	0.2877 (2)	1.06900 (19)	0.47852 (16)	0.0512 (5)	
C4	0.21460 (19)	1.10143 (15)	0.38930 (14)	0.0346 (4)	
C5	0.0702 (2)	1.00106 (18)	0.30178 (15)	0.0436 (5)	
C6	0.0001 (2)	0.86347 (18)	0.30136 (15)	0.0419 (5)	
C7	0.24792 (19)	0.58288 (15)	0.35500 (14)	0.0351 (4)	
C8	0.2303 (2)	0.5656 (2)	0.23927 (16)	0.0473 (6)	
C9	0.3764 (3)	0.55030 (19)	0.22403 (18)	0.0508 (6)	
C10	0.4458 (3)	0.5285 (3)	0.1246 (2)	0.0749 (10)	
O4	0.51673 (18)	0.76879 (19)	0.75742 (13)	0.0650 (5)	
O5	0.24957 (17)	0.72400 (14)	0.65907 (10)	0.0492 (4)	
O6	0.03386 (14)	0.74389 (13)	0.75511 (10)	0.0423 (3)	
O7	−0.1353 (2)	0.8493 (3)	0.89565 (18)	0.0976 (9)	
O8	−0.1205 (2)	0.7661 (3)	1.02988 (15)	0.0952 (8)	
O9	0.4658 (2)	0.9039 (2)	1.25864 (12)	0.0710 (6)	
O10	0.66328 (19)	0.9064 (2)	1.17615 (14)	0.0731 (6)	
N4	−0.06166 (19)	0.8072 (2)	0.96064 (14)	0.0581 (6)	
N5	0.51517 (19)	0.88887 (16)	1.17389 (13)	0.0476 (5)	
C11	0.31809 (18)	0.78573 (15)	0.85972 (13)	0.0322 (4)	
C12	0.14695 (18)	0.77861 (15)	0.85294 (13)	0.0326 (4)	
C13	0.10795 (19)	0.80780 (18)	0.95755 (14)	0.0386 (4)	
C14	0.2252 (2)	0.84029 (18)	1.06109 (14)	0.0398 (4)	
C15	0.38950 (19)	0.84785 (16)	1.06265 (13)	0.0361 (4)	
C16	0.43768 (18)	0.82182 (16)	0.96381 (14)	0.0352 (4)	
C17	0.3688 (2)	0.75745 (17)	0.75231 (14)	0.0391 (4)	
H1A	0.35880	1.24140	0.33870	0.0640*	
H1B	0.35470	1.30800	0.45420	0.0640*	
H1C	0.20820	1.27010	0.35590	0.0640*	
H2	0.26480	0.90860	0.53940	0.0600*	
H2A	0.15640	0.58830	0.48370	0.0450*	
H3	0.38410	1.13910	0.53810	0.0610*	
H5	0.02000	1.02560	0.24320	0.0520*	
H6	−0.09710	0.79410	0.24200	0.0500*	
H8	0.13930	0.56490	0.18580	0.0570*	
H10A	0.55580	0.60560	0.14430	0.1120*	
H10B	0.36680	0.52770	0.05980	0.1120*	
H10C	0.45950	0.43820	0.10560	0.1120*	
H6A	0.07860	0.73090	0.70370	0.0630*	
H14	0.19440	0.85670	1.12830	0.0480*	
H16	0.54970	0.82850	0.96720	0.0420*	

### Atomic displacement parameters (Å^2^)

**Table:**

	*U*^11^	*U*^22^	*U*^33^	*U*^12^	*U*^13^	*U*^23^
S1	0.0360 (2)	0.0322 (2)	0.0349 (2)	0.0131 (1)	0.0121 (1)	0.0137 (2)
O1	0.0460 (6)	0.0366 (6)	0.0424 (7)	0.0088 (5)	0.0010 (5)	0.0093 (5)
O2	0.0511 (6)	0.0500 (7)	0.0531 (8)	0.0239 (5)	0.0301 (6)	0.0265 (6)
O3	0.0505 (7)	0.0712 (9)	0.0659 (10)	0.0312 (7)	0.0241 (7)	0.0152 (8)
N1	0.0506 (7)	0.0353 (7)	0.0509 (9)	0.0180 (6)	0.0277 (7)	0.0199 (6)
N2	0.0475 (7)	0.0421 (7)	0.0347 (7)	0.0238 (6)	0.0164 (6)	0.0198 (6)
N3	0.0485 (8)	0.0614 (10)	0.0492 (9)	0.0275 (7)	0.0145 (7)	0.0134 (8)
C1	0.0344 (6)	0.0310 (6)	0.0310 (7)	0.0136 (5)	0.0102 (5)	0.0120 (6)
C2	0.0576 (10)	0.0412 (9)	0.0381 (9)	0.0092 (7)	−0.0054 (7)	0.0189 (8)
C3	0.0529 (9)	0.0367 (8)	0.0428 (10)	0.0026 (7)	−0.0037 (8)	0.0132 (8)
C4	0.0397 (7)	0.0318 (7)	0.0399 (8)	0.0169 (6)	0.0203 (6)	0.0156 (6)
C5	0.0465 (8)	0.0430 (8)	0.0434 (9)	0.0185 (7)	0.0060 (7)	0.0228 (8)
C6	0.0393 (7)	0.0390 (8)	0.0403 (9)	0.0115 (6)	0.0000 (6)	0.0165 (7)
C7	0.0409 (7)	0.0272 (6)	0.0368 (8)	0.0132 (5)	0.0132 (6)	0.0106 (6)
C8	0.0559 (10)	0.0513 (10)	0.0423 (10)	0.0243 (8)	0.0220 (8)	0.0198 (8)
C9	0.0598 (10)	0.0385 (8)	0.0567 (12)	0.0173 (8)	0.0324 (9)	0.0136 (8)
C10	0.0892 (17)	0.0710 (15)	0.0808 (18)	0.0340 (13)	0.0583 (15)	0.0270 (13)
O4	0.0475 (7)	0.0968 (12)	0.0513 (8)	0.0267 (7)	0.0256 (6)	0.0230 (8)
O5	0.0571 (7)	0.0597 (8)	0.0306 (6)	0.0233 (6)	0.0132 (5)	0.0160 (6)
O6	0.0395 (5)	0.0529 (7)	0.0314 (6)	0.0198 (5)	0.0039 (4)	0.0138 (5)
O7	0.0692 (10)	0.171 (2)	0.0830 (13)	0.0813 (13)	0.0218 (9)	0.0526 (13)
O8	0.0515 (8)	0.167 (2)	0.0550 (10)	0.0286 (11)	0.0271 (8)	0.0321 (12)
O9	0.0746 (10)	0.0941 (12)	0.0313 (7)	0.0259 (9)	0.0026 (7)	0.0211 (8)
O10	0.0468 (7)	0.0981 (12)	0.0612 (10)	0.0296 (8)	−0.0061 (7)	0.0238 (9)
N4	0.0382 (7)	0.0850 (12)	0.0393 (9)	0.0244 (8)	0.0082 (6)	0.0079 (8)
N5	0.0474 (8)	0.0447 (8)	0.0376 (8)	0.0141 (6)	−0.0053 (6)	0.0128 (7)
C11	0.0333 (6)	0.0298 (6)	0.0305 (7)	0.0109 (5)	0.0073 (5)	0.0098 (6)
C12	0.0348 (6)	0.0304 (6)	0.0288 (7)	0.0116 (5)	0.0041 (5)	0.0102 (6)
C13	0.0327 (7)	0.0451 (8)	0.0349 (8)	0.0158 (6)	0.0079 (6)	0.0114 (7)
C14	0.0407 (7)	0.0452 (8)	0.0294 (8)	0.0154 (6)	0.0084 (6)	0.0109 (7)
C15	0.0361 (7)	0.0346 (7)	0.0299 (7)	0.0112 (6)	−0.0004 (6)	0.0105 (6)
C16	0.0321 (6)	0.0336 (7)	0.0369 (8)	0.0124 (5)	0.0057 (6)	0.0122 (6)
C17	0.0403 (7)	0.0394 (8)	0.0365 (8)	0.0134 (6)	0.0128 (6)	0.0140 (7)

### Geometric parameters (Å, º)

**Table:**

S1—O1	1.4224 (13)	C2—C3	1.381 (3)
S1—O2	1.4276 (14)	C3—C4	1.373 (3)
S1—N2	1.6264 (16)	C4—C5	1.370 (2)
S1—C1	1.7651 (17)	C5—C6	1.378 (3)
O3—N3	1.408 (2)	C7—C8	1.408 (2)
O3—C9	1.331 (3)	C8—C9	1.351 (3)
O4—C17	1.221 (2)	C9—C10	1.490 (3)
O5—C17	1.288 (2)	C2—H2	0.9300
O6—C12	1.300 (2)	C3—H3	0.9300
O7—N4	1.210 (3)	C5—H5	0.9300
O8—N4	1.212 (3)	C6—H6	0.9300
O9—N5	1.221 (2)	C8—H8	0.9300
O10—N5	1.215 (2)	C10—H10A	0.9600
O6—H6A	0.8200	C10—H10B	0.9600
N1—C4	1.464 (2)	C10—H10C	0.9600
N2—C7	1.388 (2)	C11—C16	1.382 (2)
N3—C7	1.311 (3)	C11—C17	1.493 (2)
N1—H1B	0.8900	C11—C12	1.427 (2)
N1—H1C	0.8900	C12—C13	1.410 (2)
N1—H1A	0.8900	C13—C14	1.377 (2)
N2—H2A	0.8600	C14—C15	1.379 (3)
N4—C13	1.457 (3)	C15—C16	1.381 (2)
N5—C15	1.463 (2)	C14—H14	0.9300
C1—C2	1.380 (2)	C16—H16	0.9300
C1—C6	1.378 (2)		
			
O1—S1—O2	120.44 (8)	C8—C9—C10	133.9 (2)
O1—S1—N2	108.84 (8)	O3—C9—C8	110.33 (19)
O1—S1—C1	107.28 (8)	C1—C2—H2	120.00
O2—S1—N2	104.18 (8)	C3—C2—H2	120.00
O2—S1—C1	109.04 (8)	C2—C3—H3	120.00
N2—S1—C1	106.25 (8)	C4—C3—H3	120.00
N3—O3—C9	108.82 (18)	C6—C5—H5	120.00
C12—O6—H6A	109.00	C4—C5—H5	120.00
S1—N2—C7	124.67 (12)	C5—C6—H6	120.00
O3—N3—C7	104.87 (15)	C1—C6—H6	120.00
H1B—N1—H1C	109.00	C9—C8—H8	128.00
C4—N1—H1A	109.00	C7—C8—H8	128.00
C4—N1—H1B	109.00	H10B—C10—H10C	109.00
C4—N1—H1C	109.00	C9—C10—H10B	109.00
H1A—N1—H1B	109.00	C9—C10—H10C	110.00
H1A—N1—H1C	110.00	H10A—C10—H10B	109.00
S1—N2—H2A	118.00	H10A—C10—H10C	109.00
C7—N2—H2A	118.00	C9—C10—H10A	109.00
O7—N4—O8	123.4 (2)	C12—C11—C16	121.17 (14)
O7—N4—C13	118.79 (19)	C12—C11—C17	118.90 (14)
O8—N4—C13	117.80 (18)	C16—C11—C17	119.92 (15)
O10—N5—C15	117.66 (15)	O6—C12—C13	122.61 (15)
O9—N5—O10	123.92 (17)	C11—C12—C13	116.05 (14)
O9—N5—C15	118.42 (17)	O6—C12—C11	121.32 (14)
S1—C1—C2	121.18 (13)	N4—C13—C12	120.47 (15)
C2—C1—C6	120.80 (16)	C12—C13—C14	123.04 (16)
S1—C1—C6	118.00 (13)	N4—C13—C14	116.48 (15)
C1—C2—C3	119.21 (17)	C13—C14—C15	118.34 (15)
C2—C3—C4	119.55 (17)	N5—C15—C16	120.25 (15)
C3—C4—C5	121.35 (16)	C14—C15—C16	121.92 (15)
N1—C4—C5	118.03 (15)	N5—C15—C14	117.80 (14)
N1—C4—C3	120.61 (16)	C11—C16—C15	119.46 (15)
C4—C5—C6	119.34 (17)	O4—C17—C11	119.62 (16)
C1—C6—C5	119.70 (16)	O5—C17—C11	115.95 (16)
N3—C7—C8	112.03 (16)	O4—C17—O5	124.43 (17)
N2—C7—C8	130.75 (16)	C13—C14—H14	121.00
N2—C7—N3	117.21 (15)	C15—C14—H14	121.00
C7—C8—C9	103.93 (17)	C11—C16—H16	120.00
O3—C9—C10	115.7 (2)	C15—C16—H16	120.00
			
O1—S1—N2—C7	49.04 (16)	C2—C3—C4—N1	−177.26 (16)
O2—S1—N2—C7	178.71 (14)	C2—C3—C4—C5	1.5 (3)
C1—S1—N2—C7	−66.19 (15)	C3—C4—C5—C6	−2.1 (3)
O1—S1—C1—C2	−161.03 (14)	N1—C4—C5—C6	176.66 (16)
O1—S1—C1—C6	20.36 (16)	C4—C5—C6—C1	0.7 (3)
O2—S1—C1—C2	67.01 (16)	N2—C7—C8—C9	−179.86 (19)
O2—S1—C1—C6	−111.60 (14)	N3—C7—C8—C9	−0.7 (2)
N2—S1—C1—C2	−44.74 (16)	C7—C8—C9—O3	1.0 (2)
N2—S1—C1—C6	136.65 (14)	C7—C8—C9—C10	−179.8 (3)
N3—O3—C9—C8	−0.9 (2)	C16—C11—C12—O6	−179.60 (16)
C9—O3—N3—C7	0.4 (2)	C16—C11—C12—C13	−1.1 (2)
N3—O3—C9—C10	179.7 (2)	C17—C11—C12—O6	2.1 (2)
S1—N2—C7—N3	164.49 (14)	C17—C11—C12—C13	−179.40 (15)
S1—N2—C7—C8	−16.4 (3)	C12—C11—C16—C15	1.7 (3)
O3—N3—C7—N2	179.46 (15)	C17—C11—C16—C15	179.96 (16)
O3—N3—C7—C8	0.2 (2)	C12—C11—C17—O4	177.09 (18)
O7—N4—C13—C14	144.2 (2)	C12—C11—C17—O5	−1.9 (2)
O8—N4—C13—C12	146.3 (2)	C16—C11—C17—O4	−1.2 (3)
O7—N4—C13—C12	−34.7 (3)	C16—C11—C17—O5	179.75 (16)
O8—N4—C13—C14	−34.7 (3)	O6—C12—C13—N4	−3.2 (3)
O9—N5—C15—C14	5.4 (3)	O6—C12—C13—C14	177.93 (17)
O9—N5—C15—C16	−176.34 (18)	C11—C12—C13—N4	178.36 (17)
O10—N5—C15—C16	3.9 (3)	C11—C12—C13—C14	−0.5 (3)
O10—N5—C15—C14	−174.40 (19)	N4—C13—C14—C15	−177.38 (17)
C6—C1—C2—C3	−1.9 (3)	C12—C13—C14—C15	1.6 (3)
S1—C1—C6—C5	179.87 (14)	C13—C14—C15—N5	177.28 (17)
S1—C1—C2—C3	179.55 (14)	C13—C14—C15—C16	−1.0 (3)
C2—C1—C6—C5	1.3 (3)	N5—C15—C16—C11	−178.83 (16)
C1—C2—C3—C4	0.5 (3)	C14—C15—C16—C11	−0.6 (3)

### Hydrogen-bond geometry (Å, º)

**Table:**

*D*—H···*A*	*D*—H	H···*A*	*D*···*A*	*D*—H···*A*
O6—H6*A*···O5	0.82	1.68	2.4296 (19)	151
N2—H2*A*···O5	0.86	2.12	2.7852 (18)	134
N1—H1*A*···O4^i^	0.89	1.77	2.661 (2)	177
N1—H1*B*···N3^i^	0.89	2.24	3.041 (2)	150
N1—H1*C*···O6^ii^	0.89	2.21	3.064 (2)	160
C5—H5···O6^ii^	0.93	2.60	3.293 (2)	132
C6—H6···O8^iii^	0.93	2.60	3.176 (2)	121

Symmetry codes: (i) −*x*+1, −*y*+2, −*z*+1; (ii) −*x*, −*y*+2, −*z*+1; (iii) *x*, *y*, *z*−1.

## References

[bb1] Bernstein, J., Davis, R. E., Shimoni, L. & Chang, N.-L. (1995). *Angew. Chem. Int. Ed. Engl.* **34**, 1555–1573.

[bb2] Bettinetti, G. P., Giordano, F., La Manna, A., Giuseppetti, G. & Tadini, C. (1982). *Cryst. Struct. Commun.* **11**, 821–828.

[bb3] Bock, L., Miller, G. H., Schaper, K. J. & Seydel, J. K. (1974). *J. Med. Chem.* **17**, 23–28. 10.1021/jm00247a006 4357096

[bb4] Bruker (2004). *APEX2*, *SAINT* and *SADABS*. Bruker AXS Inc., Madison, Wisconsin, USA.

[bb5] Cason, C. J. (2004). *POV-RAY* for Windows. Persistence of Vision, Raytracer Pty Ltd, Victoria, Australia. URL: http://www.povray.org.

[bb6] Groom, C. R. & Allen, F. H. (2014). *Angew. Chem. Int. Ed.* **53**, 662–671. 10.1002/anie.201306438 24382699

[bb7] Hida, S., Yoshida, M., Nakabayashi, I., Miura, N. N., Adachi, Y. & Ohno, N. (2005). *Biol. Pharm. Bull.* **28**, 773–778. 10.1248/bpb.28.773 15863877

[bb8] Ma, M.-L., Cheng, Y.-Y., Xu, Z.-H., Xu, P., Qu, H.-O., Fang, Y.-J., Xu, T.-W. & Wen, L. (2007). *Eur. J. Med. Chem.* **42**, 93–98. 10.1016/j.ejmech.2006.07.015 17095123

[bb9] Macrae, C. F., Bruno, I. J., Chisholm, J. A., Edgington, P. R., McCabe, P., Pidcock, E., Rodriguez-Monge, L., Taylor, R., van de Streek, J. & Wood, P. A. (2008). *J. Appl. Cryst.* **41**, 466–470.

[bb10] Marques, L. L., de Oliveira, G. M. & Schulz Lang, E. (2006). *Z. Anorg. Allg. Chem.* **632**, 2310–2314.

[bb11] Maury, L., Rambaud, J., Pauvert, B., Lasserre, Y., Berge, G. & Audran, M. (1985). *Can. J. Chem.* **63**, 3012–3018.

[bb12] Nakai, H., Takasuka, M. & Shiro, M. (1984). *J. Chem. Soc. Perkin Trans. 2*, pp. 1459–1464.

[bb13] Price, C. P., Grzesiak, A. L. & Matzger, A. J. (2005). *J. Am. Chem. Soc.* **127**, 5512–5517. 10.1021/ja042561m 15826189

[bb14] Sheldrick, G. M. (2008). *Acta Cryst.* A**64**, 112–122. 10.1107/S0108767307043930 18156677

[bb15] Spek, A. L. (2009). *Acta Cryst.* D**65**, 148–155. 10.1107/S090744490804362X PMC2631630 19171970

[bb16] Subashini, A., Muthiah, P. T., Bocelli, G. & Cantoni, A. (2007). *Acta Cryst.* E**63**, o4312–o4313. 10.1107/S1600536807067190 PMC2915166 21200590

[bb17] Subashini, A., Muthiah, P. T., Bocelli, G. & Cantoni, A. (2008). *Acta Cryst.* E**64**, m250–m251. 10.1107/S1600536807067190 PMC2915166 21200590

[bb18] Westrip, S. P. (2010). *J. Appl. Cryst.* **43**, 920–925.

